# Optically active bimesogens incorporating branched central spacers†

**DOI:** 10.1039/c8ra02075b

**Published:** 2018-05-22

**Authors:** Richard J. Mandle, John W. Goodby

**Affiliations:** Department of Chemistry, University of York Heslington York YO10 5DD UK Richard.mandle@york.ac.il

## Abstract

In the current fascination with liquid crystalline dimers, bimesogens and oligomers the role of the central spacer in these systems has perhaps been somewhat neglected. In compound 1, a phenyl 4-cyanobenzoate bimesogen, the central spacer incorporates a methyl group at the 2-position and is therefore chiral. The helical twisting power of 1, measured in both 5CB and E7, was found to be 0.36 and 0.35 μm^−1^ wt%^−1^ respectively. Compound 1 exhibited a monotropic chiral nematic phase, however no twist-bend modulated phase was observed. We prepared a number of analogues of 1 incorporating different mesogenic units and observe that those with a small aspect ratio are non mesogenic, whereas those with larger aspect ratios variously exhibit chiral nematic, TB, SmC and SmB phases.

## Abstract 2

Methylene-linked liquid-crystalline dimers with chiral spacers are found to exhibit several mesophases, including the helicoidal twist-bend modulated nematic state.

## Graphical abstract

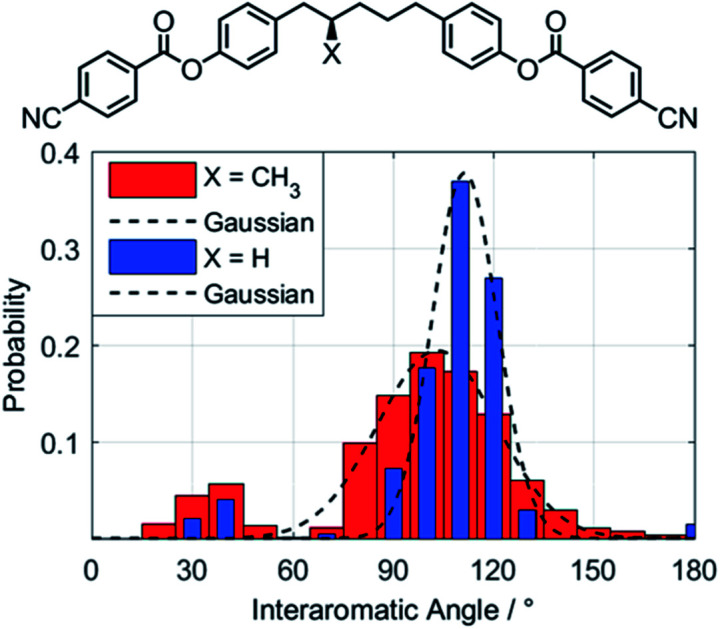

## Introduction

The twist-bend modulated nematic phase (TB), predicted by Dozov,^1^ possesses a helical structure with a pitch length of around 10 nm;^2–4^ this phase is therefore chiral despite being typically formed by achiral molecules. When formed from achiral materials the TB phase spontaneously segregates into left- and right-handed domains which can be controlled (or influenced) by external electric fields,^5^ whereas in chiral systems only a single handedness is found.^6,7^ Panov *et al.* found the TB phase to have a fast, linear electrooptic response with doubly degenerate sign,^8^ a result understood to likely be observation of the electroclinic effect^9^ and suggesting the pitch length of the TB phase is of the order of a few tens of nanometers and thus not detectable by optical means.

**Fig. 1:**
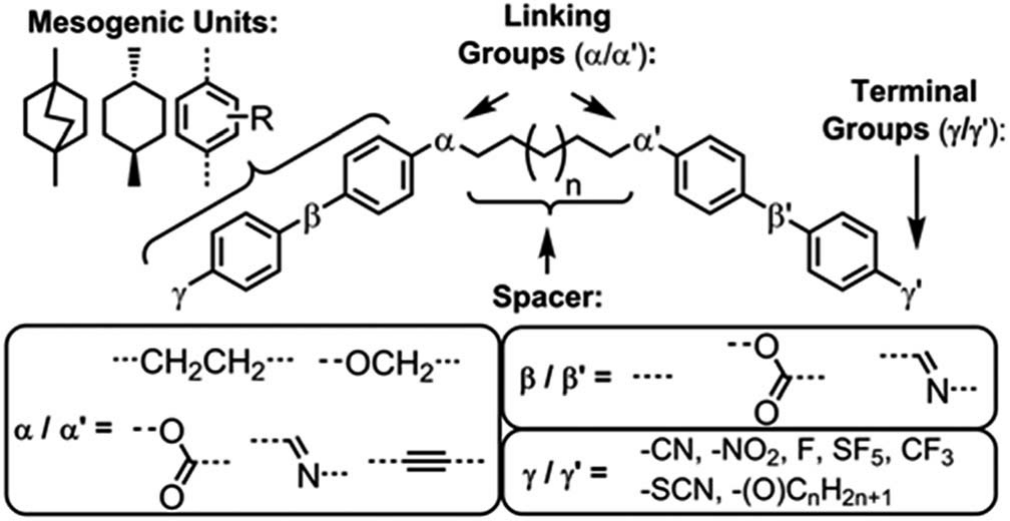
Structural variations on a simple LC dimer that have been reported to date.^28^

The TB phase exhibits a number of striking optical textures^6,10^ and has been further characterised by NMR,^11–13^ PRS,^14^ SAXS^15^ and under applied electric^16^ and magnetic fields.^17^ Transitions from the TB phase into other phases are rare, although a few examples of such transitions to smectic^18–22^ and B_6_ phases^23,24^ are known. This phase was first identified in liquid-crystalline dimers,^23,25–27^ and a relatively large number of compounds are now known to, or are suspected of, exhibiting this phase.^28,29^ As shown in Fig. 1, the relationship between molecular structure and the incidence of this phase has been an extremely active area of research in recent years; studies have investigated the role of the mesogenic units,^30^ the role of the terminal groups,^31,32^ the influence of linking groups upon bend-angle,^18,19,33–37^ the generation of the TB phase by oligomers^38–42^ and polymers.^43^ Changes to the chemical makeup of the central spacer, aside from variations in length, have yet to be studied in any great depth and this is the focus of the present study.

Herein we report on several novel dimers in which the central pentamethylene spacer contains a methyl group on the second carbon. We envisaged that the spacer would be relatively inflexible due steric interference, and may therefore confer large helical twisting power, in addition to possibly exhibiting the TB phase, which is only rarely exhibited by chiral materials.^7,44,45^

## Experimental

**Scheme 1:**
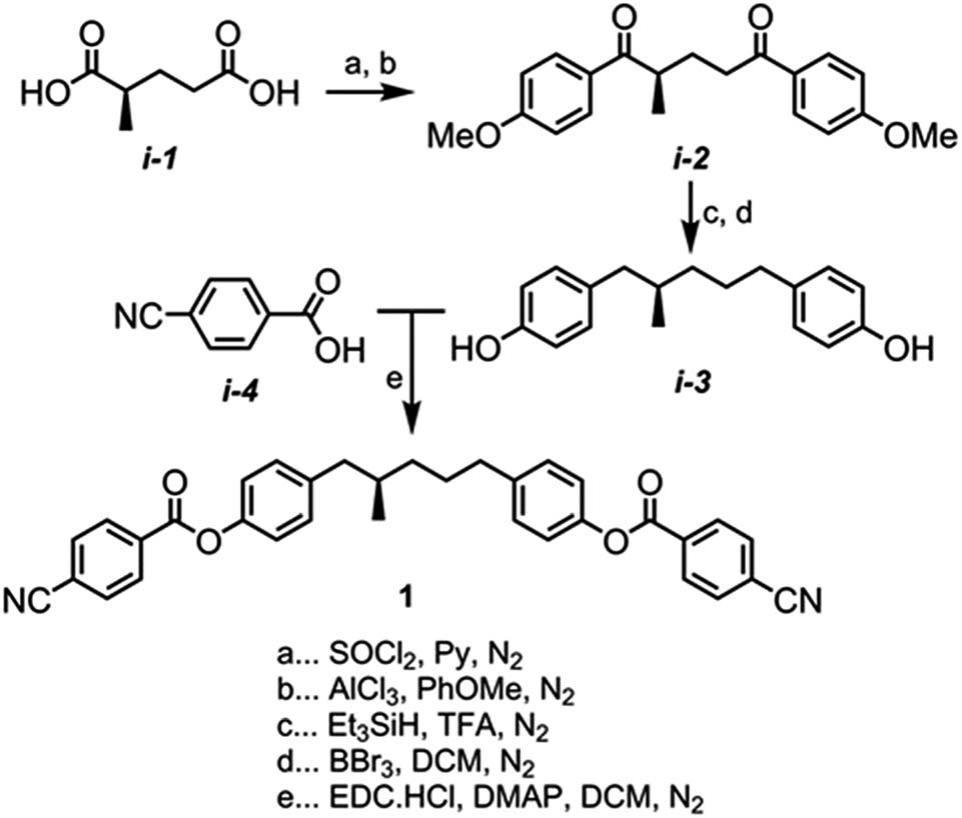


(*R*)-2-Methylglutaryl chloride was prepared by reacting the carboxylic acid, thionyl chloride, and pyridine; the crude material was used directly in Friedel–Crafts acylation of anisole with aluminium chloride to afford i-2. Following the reduction of i-2 to with Et_3_SiH/TFA the reaction mass was evaporated to dryness and used without purification in the BBr_3_ demethylation to afford i-3 in 90% yield over two steps. Finally, esterification of i-3 with 4-cyanobenzoic acid afforded compound 1 (Scheme 1). Full characterisation is given in the ESI;† the purity (>99.5%) and enantiopurity (>99%) of 1 were determined by revere-phase and chiral-phase HPLC respectively. The optical rotation of 1 was measured to be −43.9° at the sodium D line (589 nm) at 20 °C as a solution in chloroform. Compounds 2–7 were synthesised by esterification of i-3 with an appropriate carboxylic acid, all of which were available in house.

## Results and discussion

The thermal behaviour of compound 1 was investigated by polarised optical microscopy and calorimetry, transition temperatures and enthalpies are given in Table 1. Compound 1 melts at 134.1 °C and is non-mesogenic during DSC study, recrystallizing at 114.4 °C. By rapidly cooling the sample using a hotstage equipped with a liquid nitrogen pump we observe a monotropic N* phase onset temperature of circa 65 °C, this matches well with a value of 71.1 °C extrapolated from binary mixtures with 5CB. We do not observe a transition to the TB phase, even at temperatures as low as −80 °C.

**Table 1:** Transition temperatures (*T*, °C) and associated enthalpies of transition (Δ*H*, kJ mol^−1^) of compound 1, as determined by DSC at a heat/cool rate of 10 °C min^−1^

	MP	N*–Iso
*T*	134.1	71.1^a^
Δ*H*	33.2	—

a Extrapolated clearing point from binary mixtures with 5CB.

**Fig. 2:**
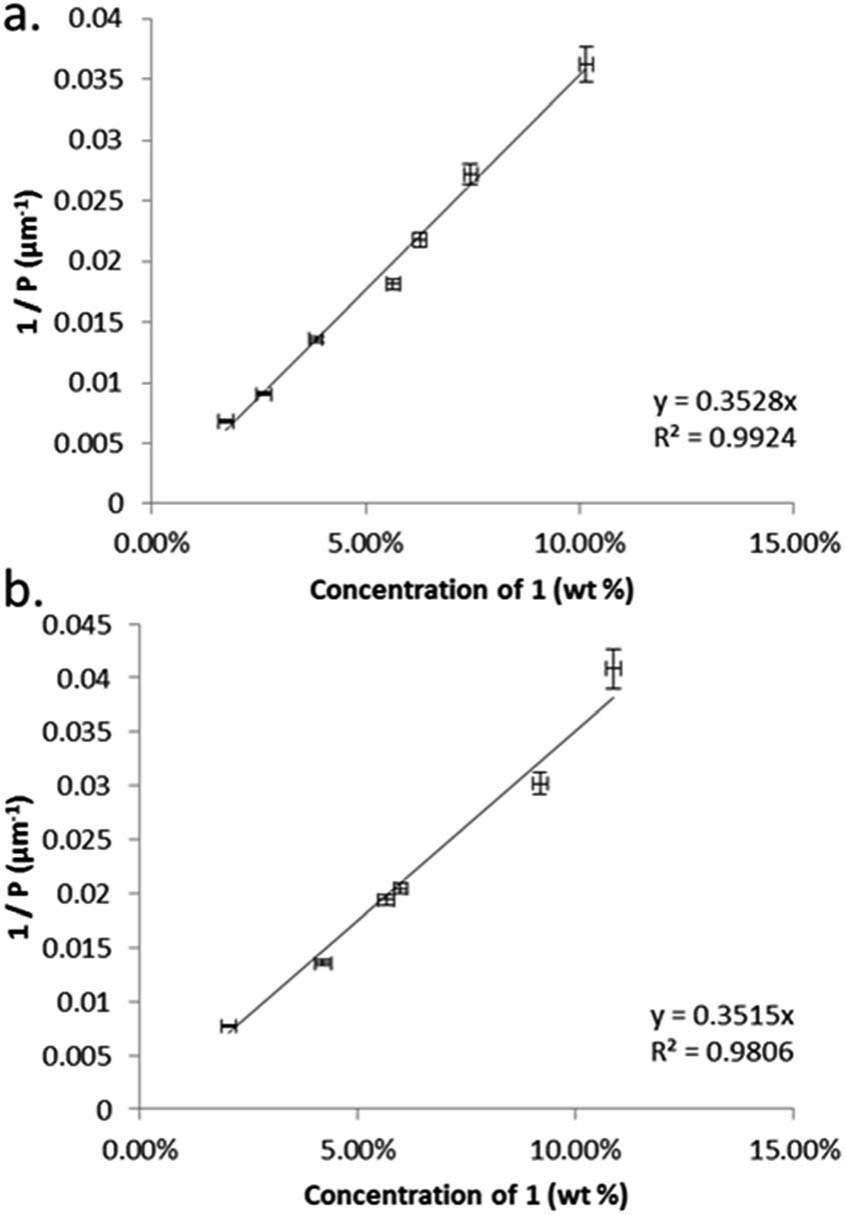
Plots of: (a) the reciprocal pitch length (μm^−1^) as a function of concentration of 1 in 5CB (mol%); (b) the reciprocal pitch length (μm^−1^) as a function of concentration of 1 in E7 (mol%).

We determined the pitch length (*P*_N*_) and helical twisting power (HTP) of 1 by the Cano wedge method using low concentration mixtures with 5CB (1.7 wt% to 10.1 wt%) and E7 (2.0 wt% to 10.9 wt%). Clearing points of each mixture were determined by DSC so as to allow each measurement of helical pitch in the N* phase to be performed at the same reduced temperature. Tabulated data are given in the ESI† to this paper. Linear fitting of the reciprocal of the pitch length (1/*P*, see Fig. 2) as a function of concentration we determine the HTP of 1 to be 0.36 μm^−1^ wt%^−1^ in 5CB and in E7. The estimated errors are ±2.44% (0.009) and ±4.1% (0.016) for 5CB and E7 respectively; the major sources of error are the wedge cells themselves (∼5%) and the measurement of pitch (up to 2%). The HTP value of 1 is low in two different hosts and the optical purity is known to be high, hence we can conclude that despite the stereogenic centre being close to both mesogenic units the transfer of chiral information is rather weak.

**Fig. 3:**
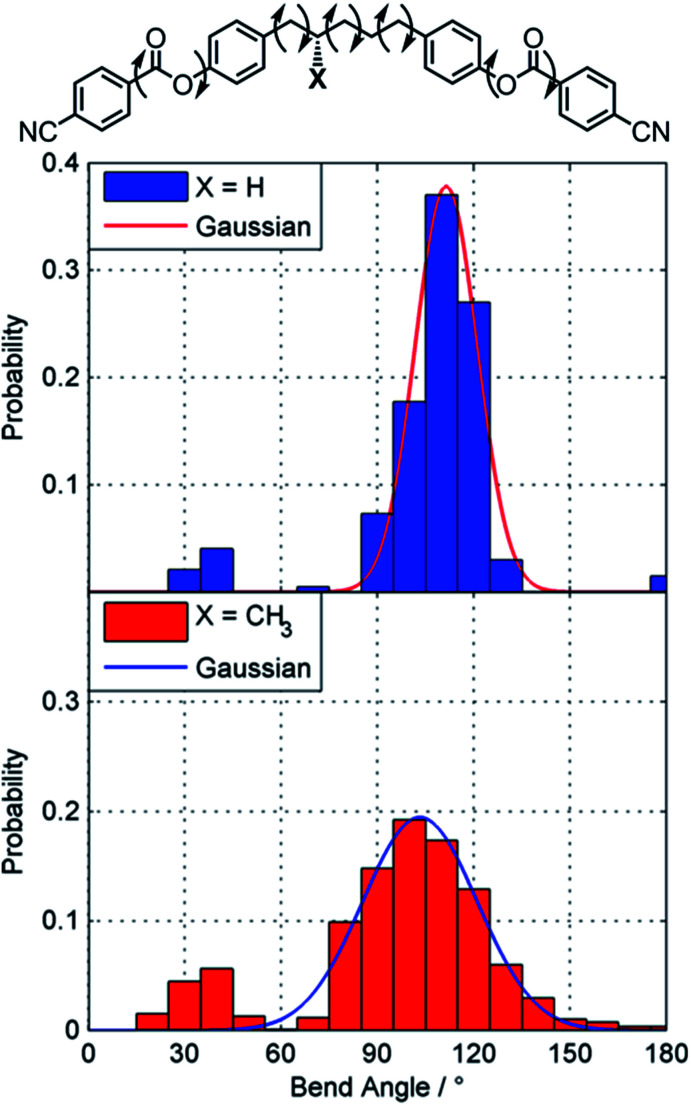
Conformational landscapes of PCB5PCB and 1 determined as described in the text. Gaussian fits are presented as a solid line.

Gray and McDonnell previously demonstrated that the pitch length decreases – and the HTP therefore increases – the closer the stereogenic centre is positioned to the mesogenic core,^46^ and so we sought to understand the present observations. Conformational studies on 1 were performed to (a) rationalise the lack of mesomorphic behaviour and (b) provide an explanation for the relatively low helical twisting power. Studies on the conformational landscape of 1 as well as that of the hypothetical compound PCB5PCB, effectively 1 without the methyl group, were performed as described by Archbold *et al.*;^47^ fully relaxed scans were performed allowing each dihedral indicated in Fig. 3 to rotate (–CH_2_–CH_2_– = threefold rotation, Ar–O and Ar–C(O) = twofold rotation). For each resulting conformer we obtain the angle between the two mesogenic units (bend angle) and the final energy which is used to obtain a Boltzmann probability for each conformer. A probability weighted average bend angle is then trivial to calculate. We apply a Gaussian fit to each histogram allowing the breadth of bend angle distribution (the FWHM) to be compared as described by Pocock *et al.*^48^

Compound 1 has a probability weighted average bend angle of 91° with a FWHM breadth of the conformer distribution is 53°. The introduction of a methyl group radically changes the conformational landscape of 1; for simple methylene-linked dimers the all *trans* form is typically the global energy minimum whereas for 1 the global minima has a single *gauche* dihedral due to steric clashes. We consider that the absence of a TB phase in 1 is probably a consequence of this broad distribution of bend angles; however the steric bulk of the lateral methyl group would also be expected to depress transition temperatures. The melting point is high due to the nitrile terminal units, a point we will return to shortly. For the hypothetical PCB5PCB – 1 without the lateral methyl group – we calculate an average bend angle of 101° with a breadth of 14°, given that the related materials CB5CB^49^ and PCB7PCB^31^ exhibit TB phases we consider it quite likely this material will also.

Previously we have shown that branched terminal chains and lateral substitution suppress LC phase formation in dimers.^50^ also noted by others,^51^ and it is probably unsurprising that this is also observed when the central spacer is branched. In terms of the impact on helical twisting power, Fig. 3 shows that compound 1 is highly flexible and can adopt a large number of conformations (and thus bend angles). High levels of molecular flexibility are generally associated with reduced helical twisting powers,^52^ and it is therefore to be expected that increasing the length of the central spacer will only reduce the HTP value further due to the increased flexibility. Similarly, our recently reported calamitic and bimesogenic materials incorporating optically active [2,2]-paracyclophanes also exhibit low HTP values due to their flexible nature.^53^

**Scheme 2:**
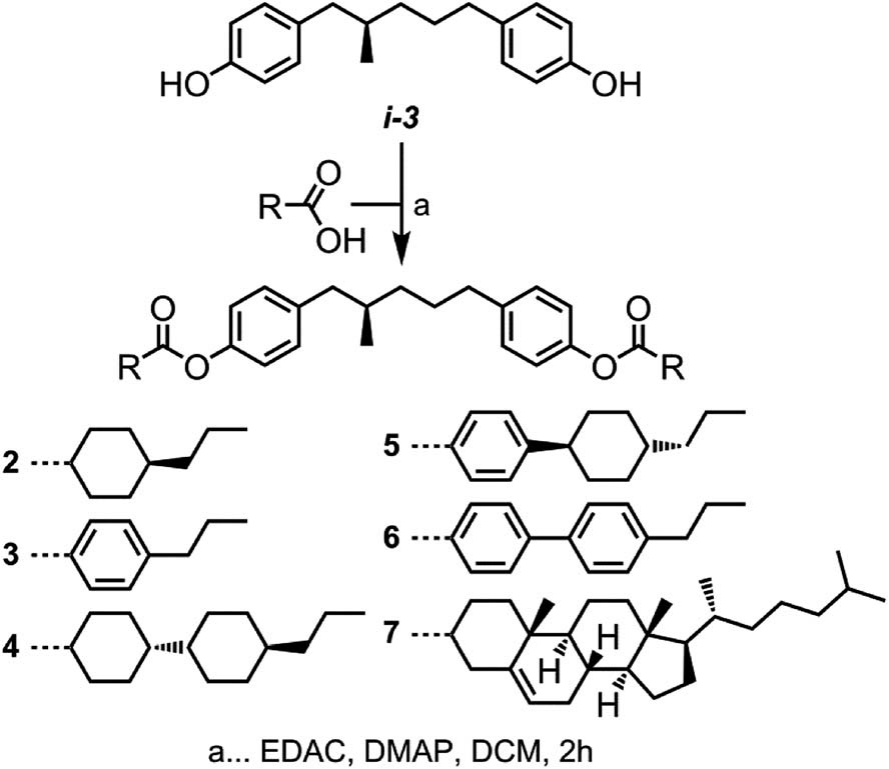


In order to reduce melting points and obtain materials that exhibit more than just transient chiral nematic phases on deep supercooling we prepared compounds 2–7 as shown in Scheme 2. Previous studies using these mesogenic units have indicated they have a tendency to exhibit a wide range of mesophases; nematic and twist-bend phases,^50^ SmA & SmB,^30^ SmC and unidentified smectic.^20^ The increased aspect ratio of compounds 4–7 was envisaged as counteracting the depression in clearing points caused by the altered conformational landscape resulting from the incorporation of a lateral methyl group. Lastly, to enable direct comparisons between materials with differing mesogenic units we used propyl-terminal chains for compounds 2–6. Transition temperatures were determined by DSC/POM and are given in Table 2.

**Table 2:** Transition temperatures (*T*, °C) and associated enthalpies (Δ*H*, kJ mol^−1^) for compounds 2–7

No.		MP	B–N*	SmA–TB	TB–N*	N*–Iso
2	*T*	61.6	—	—	—	—
	Δ*H*	40.1	—	—	—	—
3	*T*	69.1	—	—	—	—
	Δ*H*	27.1	—	—	—	—
4	*T*	66.6	169.1	—	—	233.6
	Δ*H*	34.3	9.7	—	—	2.6
5	*T*	117.1	—	110.8	123.5	219.8
	Δ*H*	37.1	—	3.5	<0.1	1.0
6	*T*	119.3	—	161.0	167.2	236.4
	Δ*H*	27.9	—	3.8	<0.1	1.8
7	*T*	123.3	—	—	—	237.4
	Δ*H*	11.4	—	—	—	1.6

The materials with the smallest aspect ratios, 1–3, were found to be non-mesogenic. Extending the length of the mesogenic units leads to mesomorphic behaviour, and so we subjected compounds 4–7 to additional study by microscopy and small- and wide-angle X-ray scattering (SWAXS). The TB phase exhibits only diffuse nematic-like scattering of non-resonant X-rays at small and wide angles,^10,26^ and thus its utility is limited to confirming there is no-electron density modulation associated with (for example) a lamellar structure. Compound 4 exhibits chiral nematic and B phases, whereas compounds 5 and 6 both exhibited the phase sequence N*–TB – SmA–B. The cholesterol derivative 7 exhibits a wide temperature range chiral nematic phase, however no latent TB phase was identified and we attribute this to the branched terminal chains which are known to suppress this phase.^32,50^

**Fig. 4:**
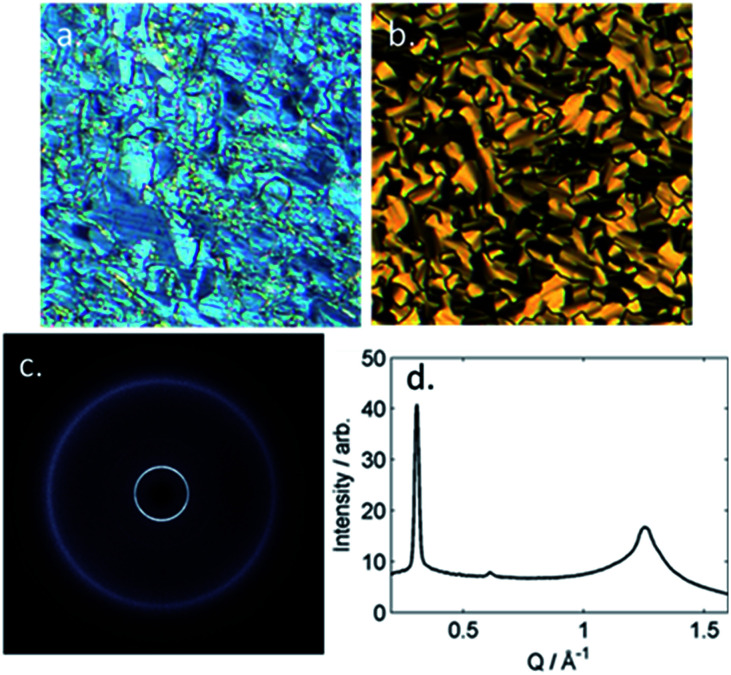
(a) Photomicrograph of the N* phase of 4 at 175 °C; (b) photomicrograph of the B phase exhibited by compound 4 at 160 °C; (c) 2D SWAXS pattern obtained for an unaligned sample of 4 in the B phase at 88 °C; (d) integrated SWAXS data (intensity *versus Q*) from the 2D pattern in (c).

The B phase of 4 was identified based on optical textures: in particular the combination of a mosaic texture (Fig. 4b) with homeotropic regions indicates a lamellar phase without tilt. When studied by SWAXS (Fig. 4c) we observe Bragg scattering at both small- and wide- angles, indicating long range order both parallel and perpendicular to the director. The layer spacing in the B phase was 20.5 Å; this compared with a molecular length of 39.1 Å (obtained at the DFT(B3LYP/6-31G(d)) level) and demonstrates the phase to be intercalated. We observed an intercalated B phase in nonamethylene linked dimers incorporating the same phenyl bicyclohexylcarboxylate mesogenic core,^30^ indicating that the branching in the central spacer is not detrimental to the incidence of this phase.

**Fig. 5:**
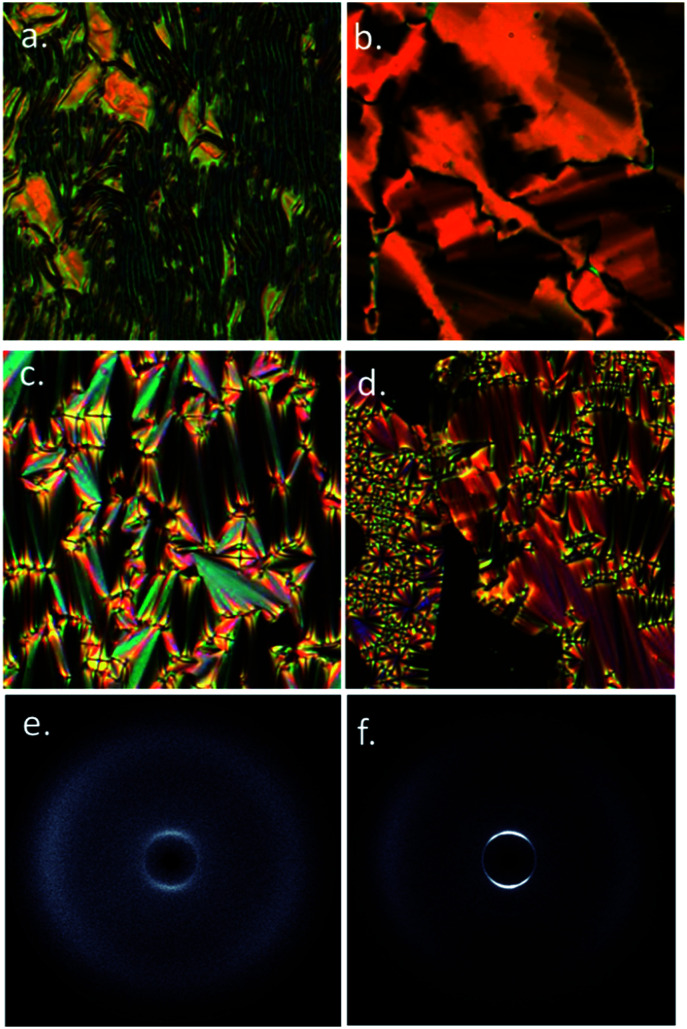
Photomicrographs of: (a) the fingerprint texture of the N* phase of 6 at 165 °C; (b) the TB phase of 6 at 162 °C; (c) the focal conic texture of SmA phase of 6 at 139 °C; (d) homeotropic and focal conic textures of the SmA phase of 6 at 144 °C. 2D SWAXS patterns of: (e) the TB phase of 6 at 163 °C; (f) the SmA phase of 6 at 152 °C.

On cooling compounds 5 and 6 from the chiral nematic phase (Fig. 5a) we obtain the blocky texture of the TB phase (Fig. 5b); further cooling yields focal-conic and fan defects (Fig. 5c) and homeotropic regions (Fig. 5d). Thus, 5 and 6 are further examples of the rare twist-bend to smectic A transition, first reported in ref. 19. We performed SWAXS on 5 and 6; the N* and TB phases lead to diffuse scattering at both small and wide angles (Fig. 5e) whereas the lamellar smectic A phase gives Bragg scattering at small angles (Fig. 5f), due to the lamellar structure. The layer spacing was determined by fitting the raw scattering data with a Gaussian function and took temperature independent values of 20.0 Å for 5 and 20.4 Å for 6 respectively. This compares with molecular lengths of 39.2 Å for 5 and 39.8 Å for 6 (both obtained at the DFT(B3LYP/6-31G(d)) level), confirming the SmA phase is interdigitated. The N*, TB and SmA phases all exhibit diffuse scattering at wide angles, this corresponds to the lateral molecular separation and confirms the lack of in-plane organisation in either phase. Aside from 5 and 6 there is only a single material known to exhibit a TB to SmA transition,^19,22^ materials generally exhibiting tilted smectic phases.^20,50^

**Fig. 6:**
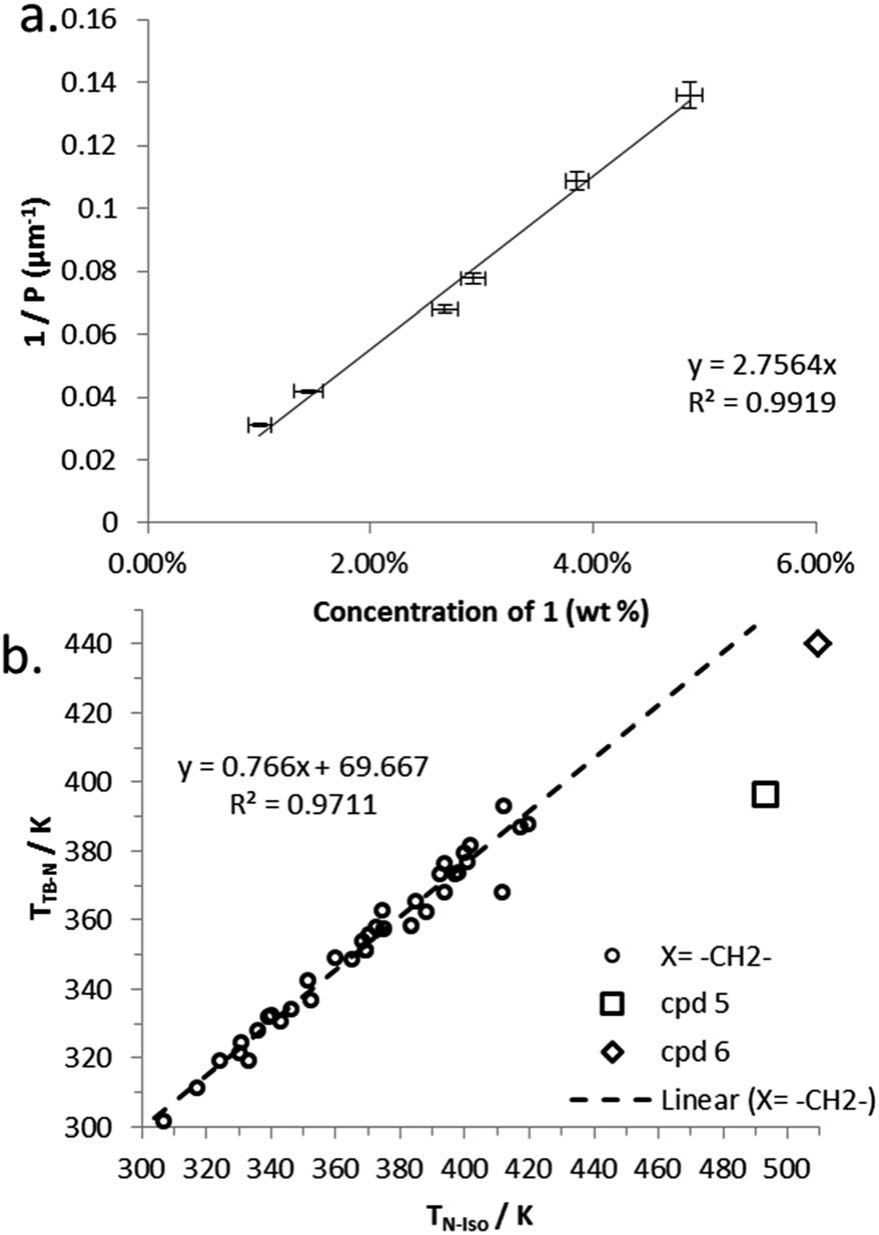
(a) Plot of the reciprocal pitch length (μm^−1^) as a function of concentration of 7 in 5CB (wt%). (b) Plot of the linear relationship between *T*_TB-N_ (K) *versus T*_N-Iso_ (K) for unbranched methylene linked dimers along with values for compounds 5 and 6, data taken from ref. 28.

Compound 7 incorporates two cholesterol derived mesogenic units in addition to the branched spacer employed in all materials in this paper. We measured *P*_N*_ and HTP of 7 by the Cano wedge method using low concentration mixtures with 5CB (1.0 wt% to 4.9 wt%). Tabulated data are given in the ESI† to this paper. Linear fitting of the reciprocal of the pitch length (1/*P*, see Fig. 6) as a function of concentration we determined the HTP of 7 to be 2.8 μm^−1^ wt%^−1^ in 5CB, with an estimated error of ±1.38% (0.038). The HTP value of 1 is low as the flexibility of the central spacer means the transfer of chiral information from the stereo centre to the mesogenic units is rather weak. As the mesogenic units of 7 are themselves chiral this leads to an order-of-magnitude increase in helical twisting power relative to 1.

Previously we have shown a linear relationship exists between the clearing point (*T*_N-Iso_) and the twist-bend to nematic phase transition (*T*_TB–N_),^54^ this is a consequence of the average molecular bend and the width of the range of bend angles exhibited by a particular class of materials.^48^ Compounds 5 and 6 appear to deviate significantly from the linear fit we reported for unbranched methylene linked dimers, as shown in Fig. 6b; this is due to the change in conformational landscape that occurs by incorporating branching groups the central spacer (Fig. 3). Increasing the aspect ratio of the mesogenic units proves to be a useful strategy generating liquid crystalline behaviour in otherwise non mesogenic bimesogens, effectively compensating for the unfavourable bend constraints imposed by the methyl group.

In terms of possible strategies for increasing values of HTP in future, positioning the stereogenic centre on the first carbon atom would be expected to give an increase, as would reducing the flexibility of the spacer. We consider that by altering the chemical makeup of the spacer it should be possible to engineer a ‘bent’ shape in a molecule with an even parity linking group (for example, introduction of 1,2-disubstituted cyclopropanes *via* the asymmetric Simmons–Smith reaction).

## Conclusions

Compound 1 is structurally related to the PCBnPCB series of dimers and bimesogens; however the central spacer incorporates a methyl group and is therefore chiral. Compound 1 exhibits a monotropic chiral nematic phase, although unlike the PCBnPCB parent series it does not exhibit the TB phase. The helical twisting power, measured in both 5CB and E7, was found to be low (0.35–0.36 μm^−1^ wt%^−1^). The origin of the relatively low HTP and lack of TB phase is due to the conformational flexibility of 1: the methyl group at the 2- position of the spacer leads to a number of steric clashes, forcing the first dihedral into a *gauche* conformation. The relatively broad distribution of bend angles favours the formation of a nematic phase over the TB phase, whereas the flexibility of the molecule means the transfer of chiral information from the stereogenic centre is rather low and thus the HTP values are low. We synthesised a number of homologues of 1; we find that using mesogenic units with a small aspect ratio (*i.e.* 2 rings) gives non-mesogenic materials, whereas larger values (3 rings) lead to significant enhancement in clearing points and two of the materials exhibiting twist-bend phases. The increased aspect ratio proves to be an effective method to compensate for the unfavourable average molecular bend imposed by the methyl group. The HTP of the cholesterol containing dimer 7 was found to be almost an order of magnitude higher than for 1, taking a value of 2.8 μm^−1^ wt%^−1^ in 5CB.

## Conflicts of interest

There are no conflicts to declare.

## Supplementary Material

RA-008-C8RA02075B-s001
