# Supplementary material for: Optically active bimesogens incorporating branched central spacers
Source: RSC Adv. 2018 May 22;8(33):18542–8. doi: 10.1039/c8ra02075b (PMC9080582; doi:10.1039/c8ra02075b)
Supplement: RA-008-C8RA02075B-s001 [file RA-008-C8RA02075B-s001.pdf]

Electronic Supplementary Material (ESI) for RSC Advances.
This journal is © The Royal Society of Chemistry 2018

1. Methods

1.1. General Techniques

Reactions were monitored by thin layer chromatography (TLC) using an appropriate solvent system. Silica coated aluminium TLC plates used were purchased from Merck (Kieselgel 60 F-254) and visualised using UV light at wavelengths of both 254 nm and 365 nm. Column chromatography was performed using flash grade silica from Fluorochem (40 - 63μm particle size). Yields refer to chromatographically (HPLC) and spectroscopically (^{1}H NMR and ^{13}C {^{1}H} NMR) homogenous material.

1.2. Nuclear Magnetic Resonance

NMR spectra were recorded on a JEOL ECS spectrometer operating at 400 MHz (^{1}H) or 100.5 MHz (^{13}C{^{1}H}) as solutions in deuterated chloroform. Spectra were referenced to the residual protic solvent for ^{1}H (7.26 ppm), ^{13}C{^{1}H} to the resonance of $CDCl_3$ (77.16 ppm)

1.3. Mass Spectrometry

Mass spectra were recorded on a Bruker compact time of flight mass spectrometer with an APCI source. We extend our gratitude to Mr. Karl Heaton of the University of York for obtaining MS data.

1.4. High Performance Liquid Chromatography

High-performance liquid chromatography was performed on a Shimadzu Prominence modular HPLC system comprising a LC-20A quaternary solvent pump, a DGU-20A$_5$ degasser, a SIL-20A autosampler, a CBM-20A communication bus, a CTO-20A column oven, and a SPO-20A dual wavelength UV-vis detector operating at 220/250 nm. The column used was an Alltech C18 bonded reverse-phase silica column (250 x 4.6 mm) with a 5 μm pore size, an internal diameter of 10 mm and a length of 250 mm and a mobile phase of chloroform/acetonitrile (9:1). Chromatograms with only one peak are quoted at >99.5 % purity.

Chiral HPLC was performed using a Daicel Chiracel OD column (250 x 2.1 mm) operated at a flow rate of 0.5 ml min^{-1} with mixtures of hexane/isopropanol as the mobile phase. The detector wavelengths were set at 230 and 250 nm respectively.

1.5. Polarised Optical Microscopy

Polarised optical microscopy was performed on a Zeiss Axioskop 40Pol microscope using a Mettler FP82HT hotstage controlled by a Mettler FP90 central processor. Photomicrographs were captured *via* an InfinityX-21 MP digital camera mounted atop the microscope. Cano wedge cells were allowed to equilibrate for 72 h at 20 °C following filling by capillary action.

1.6. Measurement of Specific Rotation

Measurements of specific rotation was performed using a Krüss Optronic manual polarimeter at the sodium D line (589 nm) at a temperature of 20 °C (measured using a standard alcohol thermometer left *in* situ in the polarimeter for 5 minutes prior to measurement) dissolved in chloroform with a path length of 10 mm. Values were compared with those calculated with DFT as described in section 1.8.

1.7. Differential Scanning Calorimetry.

Differential scanning calorimetry was performed on a Mettler DSC822^{e} fitted with an autosampler operating with Mettler Star^{e} software and calibrated before use against an indium standard (onset = 156.55 ± 0.2 °C, ΔH = 28.45 ± 0.40 Jg^{-1}) under an atmosphere of dry nitrogen.

1.8. Computational Chemistry

Quantum chemical calculations were performed using the Gaussian 09 revision e.01 suite of programmes. [4] Output files were visualised in QuteMol. [5] Generation of conformer libraries was performed by an in house Python script which is available from the corresponding author on request. A MatLab script was used to read the geometries and final energies of the output files generated during the conformational space studies.

2. Synthetic Schemes

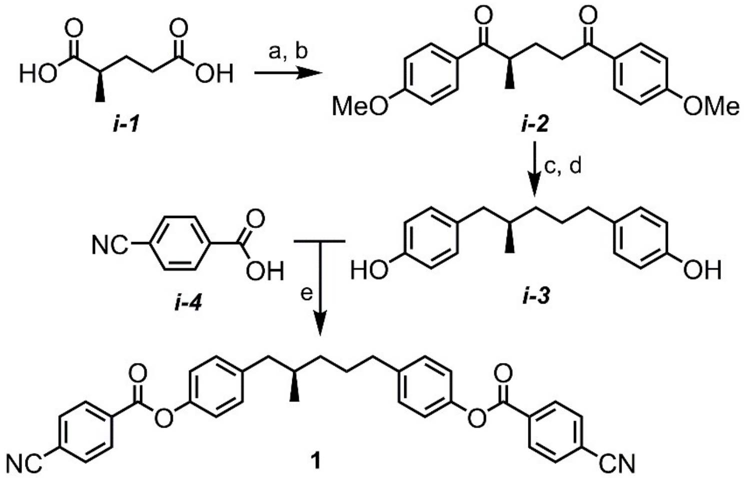

a... $SOCl_2$, Py, N_2
b... $AlCl_3$, PhOMe, N_2
c... Et_3SiH, TFA, N_2
d... BBr_3, DCM, N_2
e... EDC.HCl, DMAP, DCM, N_2

Scheme 1

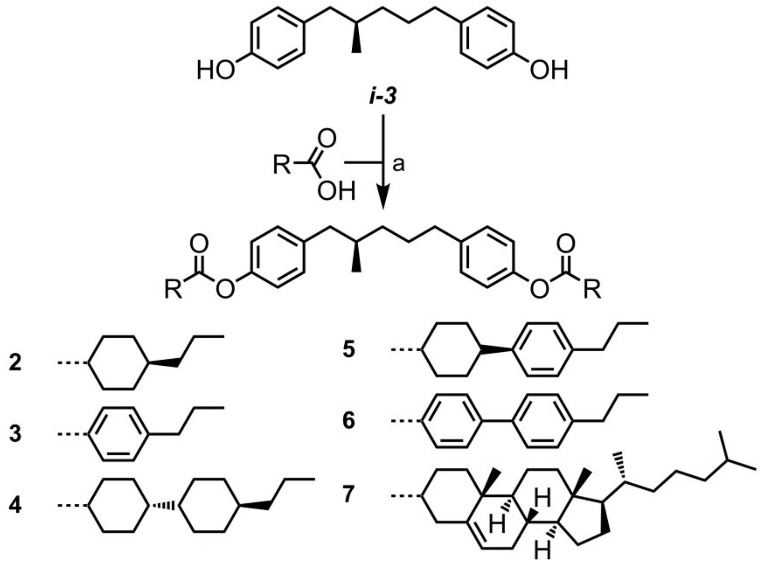

a... EDAC, DMAP, DCM, 2h

Scheme 2

3. Chemical Characterisation

3.1. Chemical Intermediates

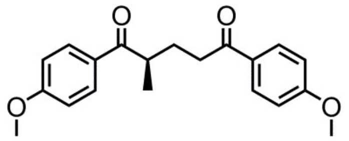

i-2 (R)-1,5-bis(4-methoxyphenyl)-2-methylpentane-1,5-dione

A suspension of (R)-2-methylglutaric acid (5 g, 34 mmol) was refluxed with thionyl chloride (30 ml) and pyridine (30 ml) for 2 h under a nitrogen atmosphere. The volatiles were removed *in vacuo* and the crude material redisolved into dichloromethane (30 ml). This solution was added dropwise to a vigorously stirred suspension of aluminium chloride (13 g, 0.1 mol) in anisole (100 ml) under an atmosphere of dry nitrogen. The suspension was stirred for 24 h before quenching with HCl (2M, 200 ml). The organic layer was separated and retained; the aqueous was washed with MTBE (2 x 100 ml) and discarded. The combined organics were dried, concentrated *in vacuo* and purified by flash chromatography with DCM as the eluent, affording the title compound as a viscous oil.

Yield: 10 g (90 %)

Rf: 0.60 (DCM)

^{1}H NMR: 1.21 (3H, t, *J* = 6.9 Hz, $\underline{H}_3$C-CH), 1.87 (1H, ddt, *J* = 6.2 Hz, *J* = 8.2 Hz, *J* = 14.1 Hz, -CH$_2$-C$\underline{H}$H-CH$_2$-), 2.23 (1H, dtd, *J* = 6.2 Hz, *J* = 7.9 Hz, *J* = 14.1 Hz, -CH$_2$-CH$\underline{H}$-CH$_2$-), 2.81 (1H, ddd, *J* = 6.4 Hz, *J* = 8.0 Hz, *J* = 16.7 Hz, C(O)-C$\underline{H}$H-CH$_2$-), 3.02 (1H, ddd, *J* = 6.4 Hz, *J* = 8.2 Hz, *J* = 16.7 Hz, C(O)-CH$\underline{H}$-CH$_2$-), 3.59 (1H, m, C(O)-C$\underline{H}$(CH$_3$)-CH$_2$-), 3.83 (3H, S, ArOC$\underline{H}_3$), 3.84 (3H, s, ArOC$\underline{H}_3$), 6.87 (2H, ddd, *J* = 2.1 Hz, *J* = 2.9 Hz, *J* = 9.0 Hz, Ar$\underline{H}$), 6.91 (2H, ddd, *J* = 2.1 Hz, *J* = 2.9 Hz, *J* = 9.0 Hz, Ar$\underline{H}$), 7.89 (2H, ddd, *J* = 2.1 Hz, *J* = 2.9 Hz, *J* = 9.0 Hz, Ar$\underline{H}$), 7.97 (2H, ddd, *J* = 2.1 Hz, *J* = 2.9 Hz, *J* = 9.0 Hz, Ar$\underline{H}$)

MS (ESI): 327.1581 (calcd. for $C_{20}H_{23}O_4$: 327.1591, M + H)

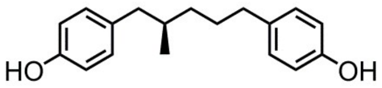

i-3: (R)-4,4'-(2-methylpentane-1,5-diyl)diphenol

Triethylsilane (24 g, 0.2 mol) was added dropwise to a solution of ***i-2*** (8.0 g, 24 mmol) in trifluoroacetic acid (100 ml) under an atmosphere of dry nitrogen. The solution was vigorously stirred for 16 h before removal of the volatiles *in vacuo*. The remaining material was redisolved in dry dichloromethane (100 ml) under an atmosphere of dry nitrogen. To this solution was then added boron tribromide (1M in hexanes, 100 ml, 0.1 mol). The reaction was monitored by TLC, complete consumption of the starting material was noted at 30 minutes. The reaction was quenched with saturated aqueous NH_4Cl (200 ml); the aqueous layer was separated and washed with DCM (3 x 100 ml) before discarding. The combined organics were dried, concentrated *in vacuo* and purified by flash chromatography with DCM as the eluent affording ***i-3*** as a viscous oil which solidified upon standing to a colourless solid.

Yield: 6.0 g (93 %)

Rf: 0.05 (DCM)

^{1}H NMR: 0.83 (3H, d, *J* = 6.6 Hz, C<u>H</u>$_3$-CH-), 1.05 – 1.18 (1H, m, -C<u>H</u>H-), 1.25 – 1.40 (1H, m, -C<u>H</u>H-), 1.50 – 1.71 (3H, m, -C<u>H</u>H-), 2.29 (2H, dd, *J* = 7.8 Hz, *J* = 13.5 Hz, Ar-C<u>H</u>H-CH(CH$_3$)-CH$_2$-), 2.45 – 2.55 (3H, m, Ar-C<u>H</u>$_2$-CH$_2$ + ArCH<u>H</u>-CH(CH$_3$)-CH$_2$-), 6.71 – 6.77 (4H, m, Ar<u>H</u>), 6.95 (2H, ddd, *J* = 1.8 Hz, *J* = 2.2 Hz, *J* = 8.8 Hz, Ar<u>H</u>), 6.99 (2H, ddd, *J* = 1.8 Hz, *J* = 2.2 Hz, *J* = 8.8 Hz, Ar<u>H</u>)

^{13}C{^{1}H} NMR: 19.50, 29.22, 35.04, 35.18, 35.75, 42.72, 115.09, 115.23, 129.53, 1300.28, 133.56, 134.85, 153.64, 153.67

MS (ESI): 271.1682 (calcd. for $C_{18}H_{23}O_2$: 271.1693, M + H)

3.2. Final Materials

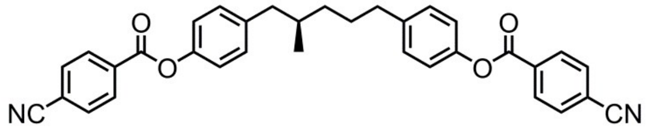

1: (R)-(2-methylpentane-1,5-diyl)bis(4,1-phenylene) bis(4-cyanobenzoate)

A solution of ***i-3*** (500 mg, 1.85 mmol), 4-cyanobenzoic acid (735 mg, 5 mmol), EDC.HCl (1.9 g, 10 mmol), and DMAP (12 mg, 0.1 mmol) in DCM (5 ml) was stirred for 0.5 h, until complete consumption of ***i-3***. The crude material was purified by flash chromatography with 1:1 DCM/hexane as the eluent, filtered through a 0.2 µm PTFE filter and finally recrystalised from ethanol/THF (10:1), affording the title compound as a white powder.

Yield: 870 mg (89 %)

Rf: 0.51

$[\alpha]_D^{293\ K}$: -43.9 °, chloroform

^{1}H NMR: 0.89 (3H, d, J = 6.7 Hz, Ar-CH_2-CH(C$\underline{H}_3$)-CH_2-CH_2-CH_2-Ar), 1.24 (1H, dddd, J = 5.2 Hz, J = 6.7 Hz, J = 10.2 Hz, J = 13.2 Hz, Ar-CH_2-CH(CH_3)-C$\underline{H}$H-CH_2-CH_2-Ar), 1.44 (1H, ddt, J = 5.2 Hz, J J = 10.2 Hz, J = 13.2 Hz, Ar-CH_2-CH(CH_3)-C$\underline{H}$H-CH_2-CH_2-Ar), 1.60 – 1.85 (3H, m, Ar-CH_2-C$\underline{H}$(CH_3)-CH_2-C$\underline{H}_2$-CH_2-Ar), 2.41 (1H, dd, J = 10.2 Hz, J = 13.2 Hz, Ar-CH$\underline{H}$-CH(CH_3)-CH_2-CH_2-CH_2-Ar), 2.55 – 2.70 (3H, m, Ar-C$\underline{H}$H-CH(CH_3)CH_2-CH_2-C$\underline{H}_2$-Ar), 7.12 (4H, ddd, J = 2.1 Hz, J = 2.4 Hz, J = 8.4 Hz, Ar$\underline{H}$), 7.20 (2H, ddd, J = 1.8 Hz, J = 2.1 Hz, J = 9.0 Hz, Ar$\underline{H}$), 7.22 (2H, ddd, J = 1.8 Hz, J = 2.4 Hz, J = 9.0 Hz, Ar$\underline{H}$), 7.80 (4H, ddd, J = 1.8 Hz, J = 2.1 Hz, J = 8.4 Hz, Ar$\underline{H}$), 8.30 (2H, ddd, J = 2.1 Hz, J = 2.4 Hz, J = 8.4 Hz, Ar$\underline{H}$)

^{13}C{^{1}H} NMR: 19.44, 29.14, 35.04, 35.65, 36.25, 43.10, 117.00, 117.98, 121.06, 121.20, 129.57, 130.31, 130.70, 132.47, 133.60, 139.69, 140.96, 148.57, 148.62, 163.79, 163.82

MS (APCI): 529.212333 (calcd. for $C_{34}H_{29}N_2O_4$: 529.212184, M + H)
561.2382 (calcd. for $C_{34}H_{28}N_2NaO_4$: 561.2382, M + Na)

Assay (HPLC): >99.5%

EE% (HPLC): >99%

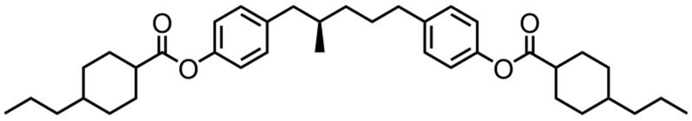

2: (R)-(2-methylpentane-1,5-diyl)bis(4,1-phenylene) bis(4-propylcyclohexane-1-carboxylate)

Quantities used: **i3** (270 mg, 1 mmol) carboxylic acid (4 mmol), EDAC (955 mg, 5 mmol), DMAP (12.2 mg, 0.1 mmol) and DCM (4 ml). The experimental procedure employed in the synthesis of **1** was followed. Column chromatography with DCM followed by recrystalisation from ethanol afforded the title compound as a white microcrystalline powder.

Yield: 480 mg (83 %)

Rf: 0.70

^{1}H NMR: 0.84 (3H, d, *J* = 6.5 Hz, Ar-CH$_2$-CH(CH$_3$)-CH$_2$-CH$_2$-CH$_2$-Ar), 0.97 (4H, qd, *J* = 3.5 Hz, *J* = 12.7 Hz, CyH), 1.15 – 1.45 (11H, m, CyH + -CH$_2$-), 1.55 (4H, qd, *J* = 3.5 Hz, *J* = 12.7 Hz, CyH), 1.60 – 1.75 (2H, m, Ar-CH$_2$-CH(CH$_3$)-CH$_2$-CH$_2$-CH$_2$-Ar), 1.80 – 1.90 (4H, m, CyH), 2.10 – 2.20 (4H, m, CyH), 2.35 (1H, dd, *J* = 8.0 Hz, *J* = 13.4 Hz, Ar-CHH-CH(CH$_3$), 2.46 (2H, tt, *J* = 3.5 Hz, *J* = 12.2 Hz, CyH), 2.55 – 2.70 (3H, m, Ar-CHH-CH(CH$_3$)CH$_2$-CH$_2$-CH$_2$-Ar), 6.93 (4H, ddd, *J* = 1.8 Hz, *J* = 2.4 Hz, *J* = 8.8 Hz, ArH), 7.10 (2H, ddd, *J* = 1.8 Hz, *J* = 2.4 Hz, *J* = 8.8 Hz, ArH), 7.14 (2H ddd, *J* = 1.8 Hz, *J* = 2.4 Hz, *J* = 8.8 Hz, ArH)

^{13}C{^{1}H} NMR: 14.49, 19.46, 20.05, 29.15, 29.19, 32.38, 35.05, 35.64, 36.23, 36.76, 39.60, 43.11, 43.77, 121.19, 121.33, 121.47, 129.33, 130.07, 138.90, 140.18, 148.90, 148.95, 175.02

MS (APCI): 575.408783 (calcd. for $C_{38}H_{55}O_4$: 575.409487, M + H)

Assay (HPLC): >99.5%

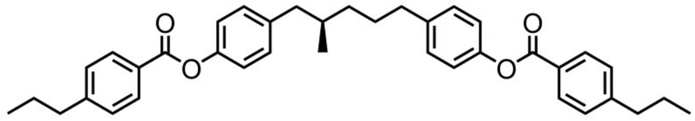

3: (R)-(2-methylpentane-1,5-diyl)bis(4,1-phenylene) bis(4-propylbenzoate)

Quantities used: **i3** (270 mg, 1 mmol) carboxylic acid (4 mmol), EDAC (955 mg, 5 mmol), DMAP (12.2 mg, 0.1 mmol) and DCM (4 ml). The experimental procedure employed in the synthesis of **1** was followed. Column chromatography with DCM followed by recrystalisation from ethanol afforded the title compound as a white microcrystalline powder.

Yield: 494 mg (88%)

Rf: 0.68

^{1}H NMR: 0.89 (3H, d *J* = 6.7 Hz, Ar-CH_2-CH($C\underline{H}_3$)-CH_2-CH_2-CH_2-Ar), 0.99 (6H, t, *J* = 7.0 Hz, Ar-CH_2-CH_2-$C\underline{H}_3$) 1.24 (1H, dddd, *J* = 5.2 Hz, *J* = 6.7 Hz, *J* = 10.7 Hz, *J* = 13.2 Hz, Ar-CH_2-CH(CH_3)-C$\underline{H}$H-CH_2-CH_2-Ar), 1.45 (1H, ddt, *J* = 5.2 Hz, *J* = 10.7 Hz, *J* = 13.2 Hz, Ar-CH_2-CH(CH_3)-C$\underline{H}$H-CH_2-CH_2-Ar), 1.58 – 1.84 (7H, m, Ar-CH_2-C$\underline{H}$(CH_3)-CH_2-$C\underline{H}_2$-CH_2-Ar + Ar-CH_2-$C\underline{H}_2$-CH_3), 2.41 (1H, dd, *J* = 8.0 Hz, *J* = 13.4 Hz, Ar-CH$\underline{H}$-CH(CH_3)-CH_2-CH_2-CH_2-Ar), 2.55 – 2.75 (7H, m, Ar-C$\underline{H}$H-CH(CH_3)CH_2-CH_2-$C\underline{H}_2$-Ar + Ar-$C\underline{H}_2$-CH_2-CH_3), 7.12 (4H, ddd, *J* = 2.1 Hz, *J* = 2.4 Hz, *J* = 8.4 Hz, Ar$\underline{H}$), 7.19 (2H, ddd, *J* = 1.8 Hz, *J* = 2.1 Hz, *J* = 9.0 Hz, Ar$\underline{H}$), 7.23 (2H, ddd, *J* = 1.8 Hz, *J* = 2.4 Hz, *J* = 9.0 Hz, Ar$\underline{H}$), 7.32 (4H, ddd, *J* = 1.8 Hz, *J* = 2.1 Hz, *J* = 8.4 Hz, Ar$\underline{H}$), 8.12 (2H, ddd, *J* = 2.1 Hz, *J* = 2.4 Hz, *J* = 8.4 Hz, Ar$\underline{H}$)

^{13}C{^{1}H} NMR: 13.89, 19.49, 24.40, 29.20, 35.08, 35.68, 36.23, 38.22, 43.15, 121.39, 121.54, 127.27, 128.79, 129.43, 130.18, 130.32, 139.08, 140.35, 149.08, 149.10, 165.50, 165.53

MS (APCI): 563.314971 (calcd. for $C_{38}H_{43}O_4$: M + H)

Assay (HPLC): >99.5%

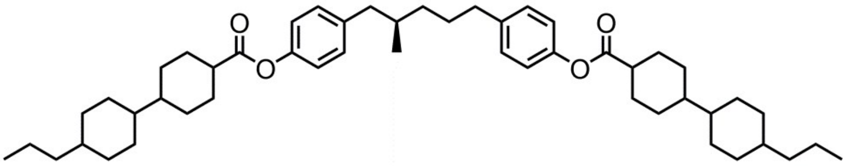

4: (R)-(2-methylpentane-1,5-diyl)bis(4,1-phenylene) bis(4'-propyl-[1,1'-bi(cyclohexane)]-4-carboxylate)

Quantities used: **i3** (270 mg, 1 mmol) carboxylic acid (4 mmol), EDAC (955 mg, 5 mmol), DMAP (12.2 mg, 0.1 mmol) and DCM (4 ml). The experimental procedure employed in the synthesis of **1** was followed. Column chromatography with DCM followed by recrystalisation from ethanol afforded the title compound as a white microcrystalline powder.

Yield: 670 mg (90 %)

Rf: 0.73

^{1}H NMR: 0.80 – 0.89 (13H, m, Cy-CH_2-CH_2-$C\underline{H}_3$ + Ar-$CH_2CH(C\underline{H}_3)CH_2$-$CH_2$-$CH_2$-Ar + Cy$\underline{H}$), 0.92 – 1.20 (20 H, m, Cy$\underline{H}$ + -CH_2-$C\underline{H}_2$-CH_2-), 1.45 – 1.90 (20H, m, Cy$\underline{H}$ + -$C\underline{H}_2$-), 2.10 – 2.20 (4H, m, Cy$\underline{H}$), 2.34 (1H, dd, *J* = 8.0 Hz, *J* = 13.5 Hz, Ar-$C\underline{H}$H-$CH(CH_3)$-), 2.43 (2H, tt, *J* = 2.1 Hz, *J* = 12.7 Hz, Cy$\underline{H}$-Ar), 2.50 – 2.60 (3H, m, Ar-$C\underline{H}$H-$CH(CH_3)$-, Ar-$C\underline{H}_2$-CH_2-CH_2-$CH(CH_3)$-), 6.93 (4H, ddd, *J* = 2.1 Hz, *J* = 2.4 Hz, *J* = 8.8 Hz, Ar$\underline{H}$) 7.08 (2H, ddd, *J* = 2.1 Hz, *J* = 2.4 Hz, *J* = 8.8 Hz, Ar$\underline{H}$), 7.13 (2H, ddd, *J* = 2.1 Hz, *J* = 2.4 Hz, *J* = 8.8 Hz, Ar$\underline{H}$)

$^{13}C\{^1H\}$ NMR: 14.57, 19.48, 20.17, 29.13, 29.27, 29.42, 30.13, 33.65, 35.01, 35.60, 36.12, 37.71, 39.92, 42.63, 43.08, 43.36, 43.81, 121.18, 121.32, 129.32, 130.06, 138.86, 140.14, 148.90, 148.95, 175.00, 175.02

MS (APCI): 739.563493 (calcd. for $C_{50}H_{75}O_4$: 739.565987, M + H)

Assay (HPLC): >99.5%

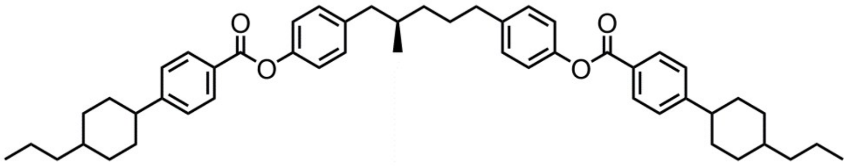

5: (R)-(2-methylpentane-1,5-diyl)bis(4,1-phenylene) bis(4-(4-propylcyclohexyl)benzoate)

Quantities used: **i3** (270 mg, 1 mmol) carboxylic acid (4 mmol), EDAC (955 mg, 5 mmol), DMAP (12.2 mg, 0.1 mmol) and DCM (4 ml). The experimental procedure employed in the synthesis of **1** was followed. Column chromatography with DCM followed by recrystalisation from ethanol afforded the title compound as a white microcrystalline powder.

Yield: 650 mg (89%)

Rf: 0.68

^{1}H NMR: 0.87 – 0.94 (9H, m, Ar-CH$_2$-CH(C<u>H</u>$_3$)-CH$_2$-CH$_2$-CH$_2$-Ar + Cy-CH$_2$-CH$_2$-C<u>H</u>$_3$), 1.00 – 1.95 (20H, m, Cy<u>H</u> + -CH$_2$-C<u>H</u>$_2$-CH$_2$ + Cy-C<u>H</u>$_2$-C<u>H</u>$_2$-CH$_3$), 2.42 (1H, dd, *J* = 7.8 Hz, *J* = 13.4 Hz, Ar-C<u>H</u>H-CH(CH$_3$)-), 2.50 – 2.65 (3H, m, Ar-C<u>H</u>H-CH(CH$_3$)…C<u>H</u>$_2$-Ar + Ar-Cy<u>H</u>), 7.07 (4H, ddd, *J* = 2.1 Hz, *J* = 2.4 Hz, *J* = 8.9 Hz, Ar<u>H</u>), 7.15 (2H, ddd, *J* = 1.8 Hz, *J* = 2.1 Hz, *J* = 8.4 Hz, Ar<u>H</u>), 7.19 (2H, ddd, *J* = 1.8 Hz, *J* = 2.1 Hz, *J* = 8.4 Hz, Ar<u>H</u>), 7.35 (4H, ddd, *J* = 1.8 Hz, *J* = 2.1 Hz, *J* = 8.9 Hz, Ar<u>H</u>), 8.05 (2H, ddd, *J* = 2.1 Hz, *J* = 2.4 Hz, *J* = 8.4 Hz, Ar<u>H</u>)

^{13}C{^{1}H} NMR: 13.42, 17.48, 18.39, 18.88, 27.78, 32.22, 32.91, 33.68, 34.22, 34.60, 35.74, 41.74, 43.60, 56.05, 120.29, 120.42, 125.99, 126.11, 128.24, 128.95, 129.10, 137.75, 139.01, 147.84, 147.84, 153.02, 164.05

MS (APCI): 727.469903 (calcd. for $C_{50}H_{64}O_4$: 727.472087, M + H)

Assay (HPLC): >99.5%

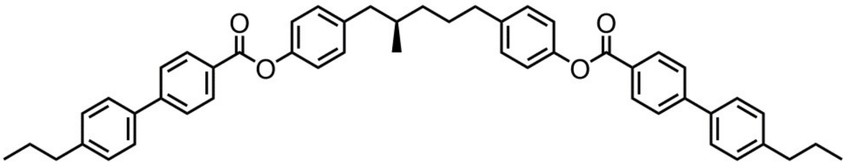

6: (R)-(2-methylpentane-1,5-diyl)bis(4,1-phenylene) bis(4'-propyl-[1,1'-biphenyl]-4-carboxylate)

Quantities used: **i3** (270 mg, 1 mmol) carboxylic acid (4 mmol), EDAC (955 mg, 5 mmol), DMAP (12.2 mg, 0.1 mmol) and DCM (4 ml). The experimental procedure employed in the synthesis of **1** was followed. Column chromatography with DCM followed by recrystalisation from ethanol afforded the title compound as a white microcrystalline powder.

Yield: 655 mg (91 %)

Rf: 0.65

^{1}H NMR: 0.89 (3H, d, *J* = 6.7 Hz, Ar-CH$_2$-CH(C$\underline{H}_3$)-CH$_2$-CH$_2$-CH$_2$-Ar), 1.24 (1H, dddd, *J* = 5.2 Hz, *J* = 6.7 Hz, *J* = 10.2 Hz, *J* = 13.2 Hz, Ar-CH$_2$-CH(CH$_3$)-C$\underline{H}$H-CH$_2$-CH$_2$-Ar), 1.44 (1H, ddt, *J* = 5.2 Hz, *J J* = 10.2 Hz, *J* = 13.2 Hz, Ar-CH$_2$-CH(CH$_3$)-C$\underline{H}$H-CH$_2$-CH$_2$-Ar), 1.60 – 1.85 (3H, m, Ar-CH$_2$-C$\underline{H}$(CH$_3$)-CH$_2$-C$\underline{H}_2$-CH$_2$-Ar), 2.41 (1H, dd, *J* = 10.2 Hz, *J* = 13.2 Hz, Ar-CH$\underline{H}$-CH(CH$_3$)-CH$_2$-CH$_2$-CH$_2$-Ar), 2.55 – 2.70 (3H, m, Ar-C$\underline{H}$H-CH(CH$_3$)CH$_2$-CH$_2$-C$\underline{H}_2$-Ar), 7.12 (4H, ddd, *J* = 2.1 Hz, *J* = 2.4 Hz, *J* = 8.4 Hz, Ar$\underline{H}$), 7.20 (2H, ddd, *J* = 1.8 Hz, *J* = 2.1 Hz, *J* = 9.0 Hz, Ar$\underline{H}$), 7.22 (2H, ddd, *J* = 1.8 Hz, *J* = 2.4 Hz, *J* = 9.0 Hz, Ar$\underline{H}$), 7.80 (4H, ddd, *J* = 1.8 Hz, *J* = 2.1 Hz, *J* = 8.4 Hz, Ar$\underline{H}$), 8.30 (2H, ddd, *J* = 2.1 Hz, *J* = 2.4 Hz, *J* = 8.4 Hz, Ar$\underline{H}$)

^{13}C{^{1}H} NMR: 14.00, 19.54, 24.65, 29.10, 35.03, 35.62, 36.06, 37.83, 43.14, 121.38, 121.52, 127.05, 127.24, 128.17, 129.22, 129.47, 130.20, 130.77, 137.27, 139.13, 140.38, 143.18, 146.25, 149.04, 149.08, 165.35, 165.38

MS (APCI): 715.376191 (calcd. for $C_{50}H_{51}O_4$: 615.378187, M + H)

Assay (HPLC): >99.5%

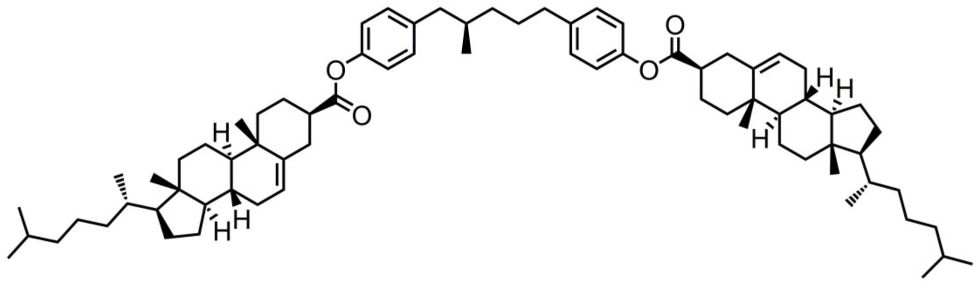

7: ((R)-2-methylpentane-1,5-diyl)bis(4,1-phenylene) (3R,3'R,8R,8'R,9R,9'R,10S,10'S,13S,13'S,14R,14'R,17S,17'S)-bis(10,13-dimethyl-17-((S)-6-methylheptan-2-yl)-2,3,4,7,8,9,10,11,12,13,14,15,16,17-tetradecahydro-1H-cyclopenta[a]phenanthrene-3-carboxylate)

Quantities used: **i3** (270 mg, 1 mmol) carboxylic acid (4 mmol), EDAC (955 mg, 5 mmol), DMAP (12.2 mg, 0.1 mmol) and DCM (4 ml). The experimental procedure employed in the synthesis of **1** was followed. Column chromatography with DCM followed by recrystalisation from ethanol afforded the title compound as a white microcrystalline powder.

Yield: 820 mg (77%)

Rf: 0.68

^{1}H NMR: 0.55 – 0.62 (4H, m), 0.75 – 2.00 (88H, m, -C<u>H</u>$_2$- + cholesterol-<u>H</u>), 2.20 – 2.50 (10H, m, Ar-C<u>H</u>$_2$), 6.84 (4H, d, *J* = 8.3 Hz, Ar<u>H</u>), 6.99 (2H, d, *J* = 8.3 Hz, Ar<u>H</u>), 7.01 (2H, d, *J* = 8.3 Hz, Ar<u>H</u>)

^{13}C{^{1}H} NMR: 11.77, 14.16, 18.63, 19.26, 19.31, 20.77, 22.49, 22.75, 23.74, 24.16, 25.16, 27.88, 28.13, 28.73, 31.65, 31.77, 34.67, 34.75, 35.24, 35.59, 35.68, 36.07, 36.81, 38.56, 39.39, 39.63, 39.77, 39.98, 40.19, 42.16, 44.54, 50.10, 56.03, 56.52, 60.01, 120.94, 121.08, 121.28, 129.09, 129.81, 138.60, 139.86, 140.51, 148.51, 148.60, 148.65, 174.16, 174.18

MS (APCI): 1063.848478 (calcd. for $C_{74}H_{111}O_4$: 1063.847688, M + H)

Assay (HPLC): >99.5%

4.1. Supplemental Figures

We present data for **1** as an exemplar, note in particular the branched central spacer leads to a much more complex ^{1}H NMR spectrum in the aliphatic region (1.0 – 2.5 ppm). In the $^{13}C\{^{1}H\}$ NMR spectrum we observe each of the six environments in the spacer exhibits a distinct resonance.

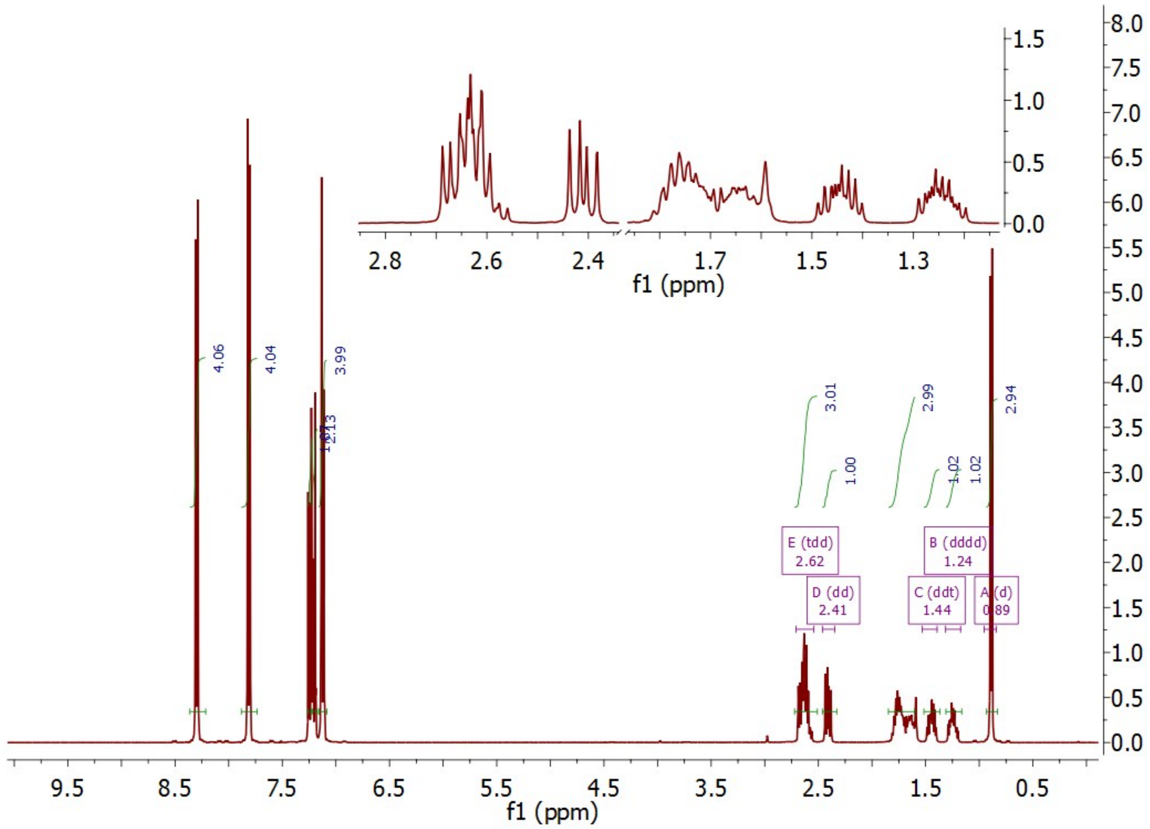

Figure SI-1: ^{1}H NMR (400 MHz, $CDCl_3$) spetra of compound **1**.

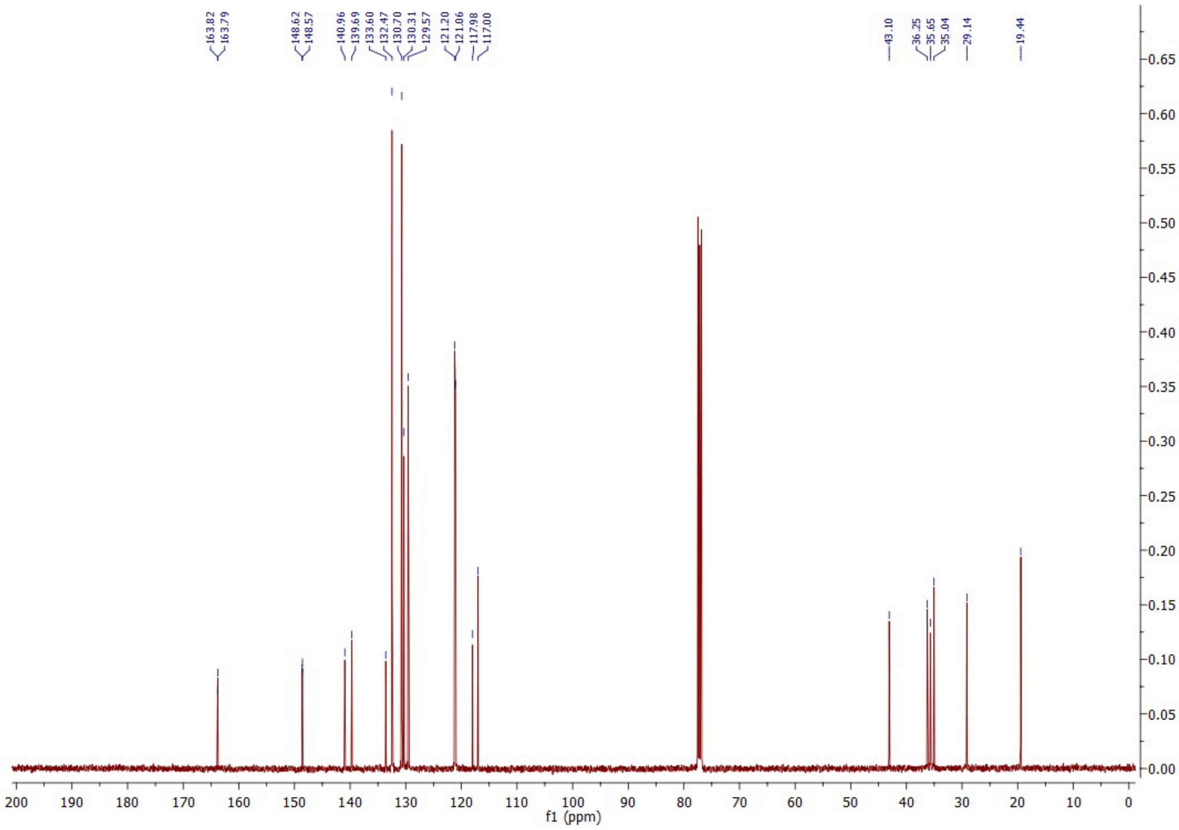

Figure SI-2: $^{13}C\{^{1}H\}$ NMR (100.5 MHz, $CDCl_3$) spectra of compound **1**.

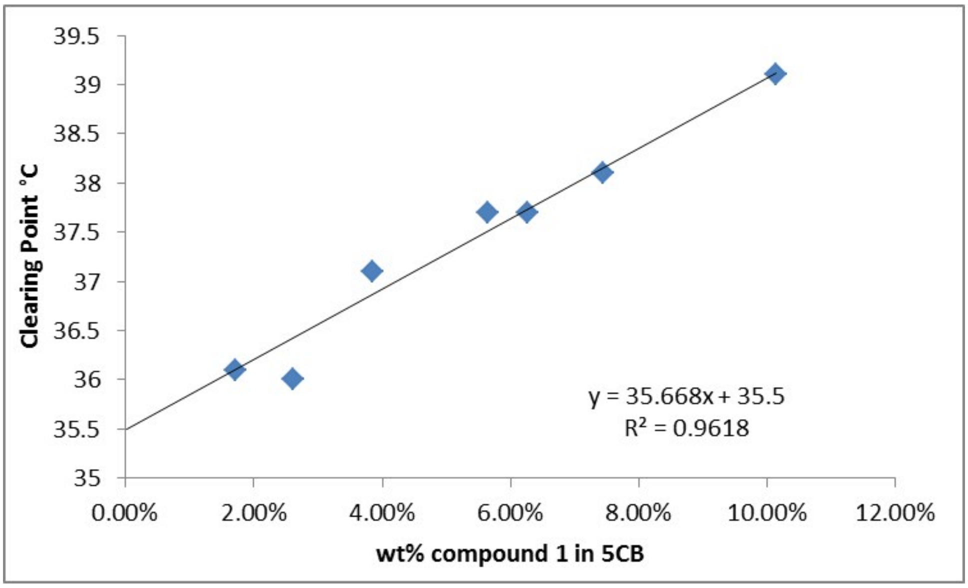

Figure SI-3: Partial phase diagram for **1** and 5CB.
